# Street Food Environment in Maputo (STOOD Map): a Cross-Sectional Study in Mozambique

**DOI:** 10.2196/resprot.4096

**Published:** 2015-08-05

**Authors:** Marcello Gelormini, Albertino Damasceno, Simão António Lopes, Sérgio Maló, Célia Chongole, Paulino Muholove, Susana Casal, Olívia Pinho, Pedro Moreira, Patrícia Padrão, Nuno Lunet

**Affiliations:** ^1^ Italian Cooperation Agency Maputo Mozambique; ^2^ Faculty of Medicine Eduardo Mondlane University Maputo Mozambique; ^3^ Faculty of Medicine – University of Porto (FMUP) Departament of Clinical Epidemiology, Predictive Medicine and Public Health Porto Portugal; ^4^ Department of Mathematics and Informatics Eduardo Mondlane University Maputo Mozambique; ^5^ Geography Department Eduardo Mondlane University Maputo Mozambique; ^6^ Faculty of Pharmacy – University of Porto (FFUP) Porto Portugal; ^7^ REQUIMTE, Laboratory of Bromatology and Hydrology University of Porto Porto Portugal; ^8^ Faculty of Nutrition and Food Sciences – University of Porto (FCNAUP) Porto Portugal; ^9^ Research Centre in Physical Activity, Health and Leisure, Faculty of Sport – University of Porto Porto Portugal; ^10^ Institute of Public Health, University of Porto (ISPUP) EPIUnit – Epidemiology Research Unit Porto Portugal

**Keywords:** fast foods, commerce, marketing, Mozambique

## Abstract

**Background:**

Street food represents a cultural, social, and economic phenomenon that is typical of urbanized areas, directly linked with a more sedentary lifestyle and providing a very accessible and inexpensive source of nutrition. Food advertising may contribute to shaping consumers’ preferences and has the potential to drive the supply of specific foods.

**Objective:**

The purpose of this study is to characterize the street food offerings available to the urban population of Maputo, the capital city of Mozambique, and the billboard food advertising in the same setting.

**Methods:**

People selling ready-to-eat foods, beverages, or snacks from venues such as carts, trucks, stands, and a variety of improvised informal setups (eg, shopping carts, trunks of cars, sides of vans, blankets on the sidewalk, etc) will be identified in the district of KaMpfumu. We will gather information about the actual food being sold through direct observation and interviews to vendors, and from the billboard advertising in the same areas. A second phase of the research entails collecting food samples to be analyzed in a specialized laboratory. The street food environment will be characterized, overall and according to socioeconomic and physical characteristics of the neighborhood, using descriptive statistics and spatial analysis. The study protocol was approved by the National Committee for Bioethics for Health in Mozambique.

**Results:**

Data collection, including the identification of street food vending sites and billboard advertising, started on October 20, 2014, and lasted for 1 month. The collection of food samples took place in December 2014, and the bromatological analyses are expected to be concluded in August 2015.

**Conclusions:**

The district of KaMpfumu is the wealthiest and most urbanized in Maputo, and it is the area with the highest concentration and variety of street food vendors. The expected results may yield important information to assess the nutritional environment and the characteristics of the foods to which a great majority of the urban population living or working in Maputo are exposed. Furthermore, this study protocol provides a framework for a stepwise standardized characterization of the street food environment, comprising 3 steps with increasing complexity and demand for human and technical resources: Step 1 consists of the evaluation of food advertising in the streets; Step 2 includes the identification of street food vendors and the characterization of the products available; and Step 3 requires the collection of food samples for bromatological analyses. This structured approach to the assessment of the street food environment may enable within-country and international comparisons as well as monitoring of temporal trends.

## Introduction

Most of the dietary changes that frequently arise from urbanization and globalization involve decreases in the consumption of foods rich in fiber such as legumes, fruits, vegetables, or whole grains and a more frequent intake of processed foods that are more likely to be energy dense and rich in sugar and salt [1]. The nutrition transition is associated with a higher frequency of noncommunicable diseases (NCDs), which are the main cause of mortality worldwide. It is estimated that by 2030, NCDs will become the most common cause of death in the African continent, surpassing the combined burden of communicable and nutritional diseases and maternal and perinatal deaths [2].

Specifically in Mozambique, a steep increase in urbanization is being observed (percentage of urban population: 21% in 1990, 31% in 2010, 36% estimated by 2050 [3]) and Western lifestyle behaviors, such as the use of processed food products (eg, sugar sweetened beverages, chicken powdered stocks), now coexist with traditional practices, as illustrated by the consumption of traditional dishes and alcoholic beverages [4,5]. Although communicable diseases are the most important contributors to the morbidity and mortality burden, NCDs are becoming more frequent and are estimated to have accounted for one-fifth of all deaths in 2010 [6].

According to the Food and Agriculture Organization of the United Nations, the term “street food” refers to a wide range of “ready-to-eat foods and beverages sold and sometimes prepared in public places, notably streets” [7]. Worldwide, 2.5 billion people eat street food every day [8], which represents a cultural, social, and economic phenomenon that is closely linked with urbanization.

Time dedicated to cooking at home has dramatically decreased among urban dwellers [9,10] and street food usually provides a very accessible and inexpensive source of energy and nutrients. A cross-sectional survey conducted in South Africa, using a nationally representative sample, found that 11% of the population consumes street food at least twice weekly [11]. Several studies have shown that street foods contribute a substantial proportion of the recommended daily allowance of energy and protein for adolescents attending school [12] and urban market women in Nigeria [13]. Moreover, these foods were shown to be fundamental for the daily diet of low-income male urban workers in Hyderabad [14], urban construction workers in Nairobi [15], and street traders in Calcutta [16].

Global influences via advertising and increased availability of imported products contribute to changes in the types of goods consumed [17,18]. A survey conducted in 2005 showed that advertising of fast food, soft drinks, and alcoholic beverages represented a relevant share of the total billboard advertising in the city of Maputo, suggesting the need for assessing the influence of advertising on food availability and dietary habits.

In countries under epidemiological transition, the characterization and monitoring of trends in the street food environment, and specifically the nutritional profile of street food, is particularly important in the context of the efforts for prevention of NCDs. Nevertheless, at present, there is little research on the street food environment in Mozambique. The purpose of this study is to characterize the street food offerings available to the urban population of Maputo and the billboard food advertising in the same setting. The specific aims are: (1) to characterize and map the spatial distribution of street food vendors and food advertising in the city of Maputo; (2) to describe the nutritional composition of the food sold in the streets; and (3) to classify the foods being sold according to the extent and purpose of their processing, such as unprocessed/minimally processed foods, processed ingredients, and ultra-processed food products.

## Methods

### Overview

This project will comprise a survey for identification and mapping of street food vendors, sampling of street foods and assessment of the billboard food advertising, in Maputo, Mozambique.

The protocol was approved by the National Committee for Bioethics for Health in Mozambique (Comité Nacional de Bioética para a Saúde, Ref. 223/CNBS/14).

### Street Food Vendors

#### Inclusion and Exclusion Criteria

People selling ready-to-eat food, beverages, or snacks from any venue other than a permanent storefront business or established farmers market are potentially eligible for the study. We will select carts, trucks, stands, and a variety of improvised informal setups (eg, shopping carts, trunks of cars, sides of vans, blankets on the sidewalk, people with coolers on the side of the road, etc), as well as “in-transit” street food vendors.

The exclusion criteria are the following: (1) food establishments with 4 permanent walls; (2) permanent storefront business; (3) street vendors selling exclusively nonfood products or raw foods not ready-to-eat; (4) street vendors operating in closed public spaces (ie, markets) or organized entities (ie, farmers markets, food fairs); and (5) food stalls and carts that are part of permanent stores or licensed establishments.

#### Recruitment Plan and Study Design

The administrative repartition of the city of Maputo is made up of 7 districts: KaMpfumu, Nlhamankulu, KaMaxaquene, KaMavota, KaMubukwana, KaTembe, and KaNyaka. The current study will be limited to the district of KaMpfumu, which is the wealthiest and most urbanized [19] among the municipal districts and is considered to represent the area with the highest concentration and variety of street food vendors.

KaMpfumu is composed of 11 neighborhoods: Bairro Central A, B, and C; Alto Maé A and B; Malhangalene A and B; Polana Cimento A and B; Coop; and Sommerschield. Field researchers will canvass specific portions of these neighborhoods and assess the presence of street food vendors in all publicly accessible roads.

Our sampling procedure starts with the identification of all the public transport stops present in the KaMpfumu district (n=134). Among them, we will randomly select 30 stops—of which, only 20 stops will be actively explored; the rest will be made available if the desired number of vendors to be interviewed is not met using the initial group. For each stop we will draw a 500-meter buffer to identify the study area. We will exclude those portions of buffers that fall outside the administrative borders of the district and, in order to avoid sampling the same area more than once, we will treat the overlapping of 2 or more buffers as 1 whole. The actual study area will be delimited using natural borders (ie, sea, mountains) and main avenues/streets.

The public transport stops distribution, which includes *chapas* (private vans) and public buses, was assessed in 2012 as part of a bigger project of formulation of a “Comprehensive Urban Transport Master Plan for Greater Maputo” undertaken by the government of Mozambique with the assistance of the government of Japan [20]. The rest of the maps have been produced by the Maputo Municipal Council (Conselho Municipal Cidade de Maputo) through the interpretation and digitalization (scale 1:5,000) of a 2012 aerial map with high resolution (2m) [19].

#### Data Collection

Field researchers will operate in pairs, canvassing the neighborhoods in search of street food vendors. Each pair will walk through each publicly accessible street in the selected area and, when a vendor is identified, investigators will mark the position on the map and approach the vendor. A similar methodology has already been field-tested in a previous research on street food vendors in the Bronx borough of New York City [21]. The interviews will be carried out daily, during working days, from 9:00 am to 4:00 pm.

Street food vendors will be asked if they agree to collaborate in the data collection and to participate in a short 10- to 15-minute interview. In the case of a positive response, the researchers will carry out the questionnaire immediately or at a later time more convenient for the vendor.

In addition to assessing the business’ operating hours and location, researchers will gather information about the actual food being sold. They will observe and take note of the type of food products available, the size of portions, the price, and the types of food packages. The vendors will be specifically asked about the date and place of preparation, storage and packaging characteristics, and provenance of water being sold.

To prevent vendors from being interviewed twice, the questionnaire starts with a control question asking if the vendor has already been interviewed. Furthermore, after the completion of each interview, each vendor is given a sticker with the logo and Web site of the research project and is asked to put it on the box or cart used to sell products to signal to other interviewers that the vendor has already participated in the study.

Whenever the seller does not agree to participate in the data collection, or when the approach is not feasible—particularly with mobile vendors who are “in-transit”—researchers will still record the geographical position and any other relevant information that can be gathered about their activity, based on the observation of their vending site and of the products available.

For those ready-to-eat foods that are not industrially processed—which means they are either cooked and sold on the street or home-cooked and then sold on the street—a standardized recipe will be used as reference to estimate their nutritional composition. Previous nutritional studies will provide specific information for the Mozambican context [5].

The criteria defined by Monteiro et al [22] will be used to classify foods according to the extent and purpose of their processing into 3 groups: (1) unprocessed/minimally processed foods, (2) processed ingredients, and (3) ultra-processed food products.

Once a map of street vendors is completed, the second phase of the research entails collecting food samples to be analyzed for nutritional composition in a specialized laboratory. Only the most common home-cooked foods will be considered—sold whenever possible in at least 4 different vending sites—up to a maximum of approximately 25 different dishes.

A sample of each food, corresponding to 1 unit or the usual dose, will be bought from 4 different, randomly selected sites among the street vendors previously interviewed. Samples will be properly stored (cold chain) until the bromatological analyses are conducted.

Before analysis, samples will be defrosted, total weight compared to detect moisture losses during storage and shipping, and immediately analyzed for moisture. All determination will be performed at least in duplicate. The following analyses will be conducted:

1. Moisture analysis will be performed by oven drying at 103°C until constant weight [23];
2. Total fat and protein contents will be determined by the Soxhlet and the Kjeldahl procedures, respectively, while total carbohydrates plus fiber will be estimated by the difference [23];
3. Cholesterol and fatty acid analyses will be evaluated on the same lipidic extract, after acid hydrolysis, and using normal phase high performance liquid chromatography for cholesterol and gas chromatography for the fatty acid ethyl esters, as validated by Cruz et al [24]. The distinction between *cis* and *trans* fatty acids will be included. For fried dishes, total polar compounds in the extracted lipids will be quantified by size exclusion high performance chromatography [25] in order to detect the degree of oil heat abuse;
4. Sodium and potassium content analysis (total Na+ and K+) will be quantified by flame photometry, using the method of Vieira et al [26]. Salt content will also be assessed using a mobile technology (B-721 LAQUAtwin Compact Salt Meter, Horbia, Tokyo, Japan) based on the ion electrode method [27].

Results will be expressed on a fresh mass basis, both per 100 g and per dose, based on the mass sold as individual dose by each individual vendor.

In the selection of the foods to be analyzed, priority will be given to those that are most commonly available and that are more representative of the typical home-cooked foods sold in the streets.

The food sampling was defined in such a way as to account for the expected diversity and variability of home-cooked foods among street vendors. However, no statistical analysis requiring a specific sample size was defined in advance, as this is essentially an exploratory analysis whose main purpose is to complement the data already available on the composition of home-cooked foods sold in the street.

#### Data Analysis and Sample Size

**Figure 1:**
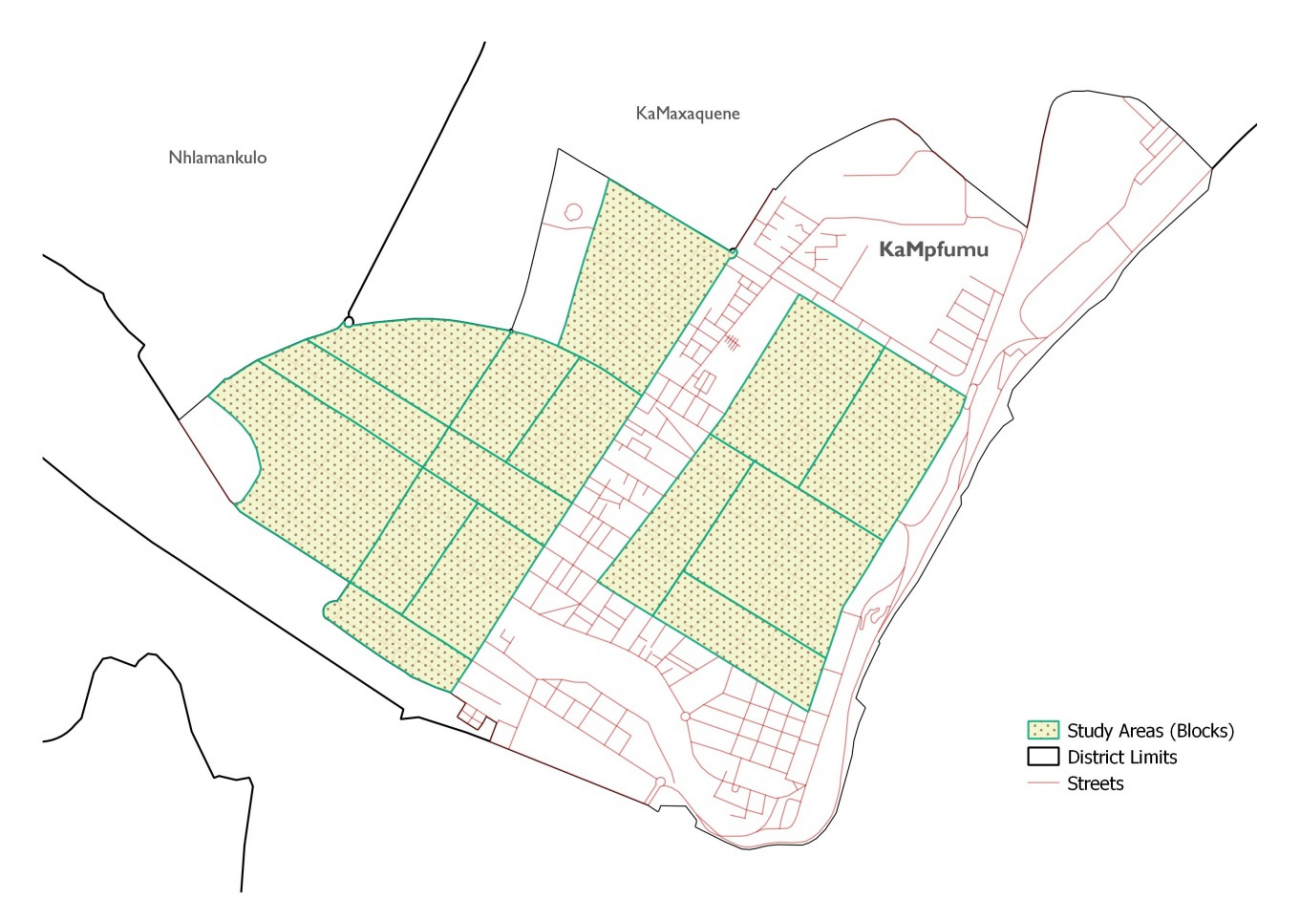
Study areas in the district of KaMpfumu, Maputo. Source: Ministry of Land and Urban Planning.

The study area will be divided into 15-block areas (Figure 1). For each area we expect to interview approximately 30 street vendors, up to a total of approximately 450 vendors.

The street food environment will be characterized—overall and according to socioeconomic and physical characteristics of the neighborhood [19]—using descriptive statistics and spatial analysis. Precision estimates will be computed taking into account the design effect due to cluster sampling.

Assuming a design effect up to 1.1, a sample size of approximately 450 will yield 95% confidence intervals up to 10% wide for observed proportions ranging between approximately 30% and 70%, and 95% confidence intervals for means with a width of approximately 20% of the observed standard deviation.

Training of the interviewers and use of standardized procedures for data collection is expected to contribute to a low proportion of missing data, and no imputation is being planned.

#### Ethical Considerations

The risks associated with participation in this study are minimal, they do not constitute a threat to confidentiality and are not expected to harm or disrupt the vendors’ businesses.

Subjects may experience discomfort and nervousness to participate because of concerns related to investigations from regulatory authority, and this could be the main reason for refusal in participating. To minimize the risk that researchers could be perceived as agents of regulatory authorities, we will utilize residents of the neighborhoods to collect data. Moreover, we will make use of younger students who would unlikely be perceived as a threat.

To reduce the risk of interfering with vendors’ businesses, when necessary, we will make attempts at interviewing them at different times of the day (ie, outside of rush hours) as long as this does not conflict with the research objectives. The questionnaire that will be used for field data collection is estimated to require around 10-15 minutes to complete, and most of the information will be gathered by simply observing the vending site without the need of constantly interacting with the vendor.

No data referring to human subjects will be collected, as the unit of the analysis is not the person selling but the vending site. The only information that will be collected refers to the products being sold and the characteristics of the vending site. Particularly, most of this type of information could be easily obtained just by observing the vending site and asking those same questions about price or provenance, for example, that clients normally ask when purchasing a good/service.

A waiver to the requirements for informed consent is reputed as necessary, given the particular conditions of the present study: no human data or any personally identifiable information is being recorded; the research involves no more than minimal risk to the participants; the waiver will not adversely affect the rights and welfare of the participants; the research could not be practicably carried out without the waiver; and participants will be informed about the research objectives and will be able to opt out of the study at any time.

### Billboard Advertising

#### Inclusion and Exclusion Criteria

Fixed billboards with outdoor static advertising are eligible for the study, regardless of their size. We will select all permanent billboards located in any of the streets canvassed by field researchers when aiming to identify street food vendors.

The exclusion criteria are the following: (1) mobile advertising (eg, on vans or selling carts); (2) advertising screens; (3) sponsored equipment (eg, sun-umbrella, coolers, chairs); and (4) promotional printed material (eg, flyers, menus).

#### Study Design and Data Analysis

Field researchers will record the location and take photographs of all the billboards observed while canvassing the different neighborhoods of the KaMpfumu district of Maputo. The billboards will then be described regarding their size, format, and content, including the use of marketing techniques with special appeal to children and adolescents (eg, cartoon characters, animation, celebrities, sports personalities) [28] and the food items advertised (eg, alcoholic beverages, including all types of beverages containing alcohol; soft drinks; fast food, including both pre-cooked and ready-to-eat meals; and non-fast food, including all types of food items not considered in the other groups).

Data analysis will comprise the description of the location of the billboards and their physical characteristics and content. The analyses will take into account, for example, the proximity of billboards to schools and youth recreational sites, as well as the assessment of their target audience through content analysis. No minimum sample size was defined since the primary objective of this section is mostly descriptive and thus its informative value is not exclusively dependent on the number of billboards.

## Results

Data collection, including the identification of street food vending sites and billboard advertising, as well as their characterization by questionnaire or observation, started on October 20, 2014, and lasted for 1 month. The collection of food samples took place in December 2014, and the corresponding bromatological analyses are expected to be concluded in August 2015.

## Discussion

The knowledge and insights gained from this study will potentially advance research efforts surrounding nutrition in the urban context and ultimately may lead to better prevention of diet-related NCDs (eg, obesity, cardiovascular diseases, malnutrition) and more suitable interventions (eg, food regulations and dietary guidelines). The expected results will provide important information on the nutritional environment and the characteristics of the foods to which a great majority of the urban population of Maputo is exposed, though generalization to other urban settings in Mozambique may not be possible.

However, this study protocol provides a framework for a stepwise standardized characterization of the street food environment, comprising 3 steps with increasing complexity and demand for human and technical resources: Step 1 consists of the evaluation of food advertising in the streets; Step 2 includes the identification of street food vendors and the characterization of the products available; and Step 3 requires the collection of food samples for bromatological analyses. This structured approach to the assessment of the street food environment may enable within-country and international comparisons as well as monitoring of temporal trends.

The project is presented to the general population in a Web site [29], where the main results will also be presented, in addition to the submission for publication in international peer-reviewed journals and scientific meetings. A report will be prepared for presentation of the main results to the local authorities.

## Appendix

Multimedia Appendix 1 Comments from funding agency's reviewer #1.

Multimedia Appendix 2 Comments from funding agency's reviewer #2.

## Abbreviations

NCD: noncommunicable disease
