# Supplementary material for: Street Food Environment in Maputo (STOOD Map): a Cross-Sectional Study in Mozambique
Source: JMIR Res Protoc. 2015 Aug 5;4(3):e98. doi: 10.2196/resprot.4096 (PMC4705368; doi:10.2196/resprot.4096)
Supplement: Multimedia Appendix 1 [file resprot_v4i3e98_app1.pdf]

Revision of the project: A cross-sectional analysis to assess the street food environment in the city of Maputo

Checklist of items that should be considered in the review of the research project

1) Relevance of the objectives

The study objectives are well described and relevant both on a nutrition and a public health level.

2) Quality of the study design
   The study is well described.
   A key issue remains sampling, for any inference purpose. This is in any case adequately addressed,

3) Appropriateness of the budget
   The budget is acceptable.

4) Characteristics and composition of the research team
   The applicant group and research team have documented experience on public health studies in Mozambique, and is internationally recognized in the field. The composition of the research team is adequate.

5) Recommendation for funding (yes/no)
   yes

6) Possible implications for public policies
   The project has clear and potentially relevant implications for public (health) policies.

7) Additional comments/recommendations
   This is well presented, original study, with a reasonable budget, and should therefore be supported.
