# Supplementary material for: Street Food Environment in Maputo (STOOD Map): a Cross-Sectional Study in Mozambique
Source: JMIR Res Protoc. 2015 Aug 5;4(3):e98. doi: 10.2196/resprot.4096 (PMC4705368; doi:10.2196/resprot.4096)
Supplement: Multimedia Appendix 2 [file resprot_v4i3e98_app2.pdf]

Revision of the project: A cross-sectional analysis to assess the street food environment in the city of Maputo

Checklist of items that should be considered in the review of the research project

1) Relevance of the objectives

   The objectives are very relevant at public health level. IN fact, the study results would allow to map the distribution of street food selling in Maputo, in order to advice the policy makers on the potential impact of such new food on health.

2) Quality of the study design
   The study is well described and the timeline coherent with the goals.

3) Appropriateness of the budget
   The budget is indeed acceptable.

4) Characteristics and composition of the research team
   The group has large experience on public health research, including Mozambique.
   The composition of the research team is properly dimensioned respect to the three study aims.

5) Recommendation for funding (yes/no)
   yes

6) Possible implications for public policies
   Public health policies would definitely be influenced by the knowledge of presence and local distribution of food street environments in Mozambique. A larger study should, however, assess the actual extent of such consumption from the local population at individual level.

7) Additional comments/recommendations
   This an original study that therefore be funded either for the strength of the team and the clear study design, and for the ultimate goals.
